# An Efficient hybrid filter-wrapper metaheuristic-based gene selection method for high dimensional datasets

**DOI:** 10.1038/s41598-019-54987-1

**Published:** 2019-12-09

**Authors:** Jamshid Pirgazi, Mohsen Alimoradi, Tahereh Esmaeili Abharian, Mohammad Hossein Olyaee

**Affiliations:** 1 Faculty of Engineering, Department of Computer Engineering, University of Gonabad, Gonabad, Iran; 2 Faculty of Electronic, Computer & IT Department of Computer, Qazvin Islamic Azad University, Qazvin, Iran

**Keywords:** Machine learning, Microarrays

## Abstract

Feature selection problem is one of the most significant issues in data classification. The purpose of feature selection is selection of the least number of features in order to increase accuracy and decrease the cost of data classification. In recent years, due to appearance of high-dimensional datasets with low number of samples, classification models have encountered over-fitting problem. Therefore, the need for feature selection methods that are used to remove the extensions and irrelevant features is felt. Recently, although, various methods have been proposed for selecting the optimal subset of features with high precision, these methods have encountered some problems such as instability, high convergence time, selection of a semi-optimal solution as the final result. In other words, they have not been able to fully extract the effective features. In this paper, a hybrid method based on the IWSSr method and Shuffled Frog Leaping Algorithm (SFLA) is proposed to select effective features in a large-scale gene dataset. The proposed algorithm is implemented in two phases: filtering and wrapping. In the filter phase, the Relief method is used for weighting features. Then, in the wrapping phase, by using the SFLA and the IWSSr algorithms, the search for effective features in a feature-rich area is performed. The proposed method is evaluated by using some standard gene expression datasets. The experimental results approve that the proposed approach in comparison to similar methods, has been achieved a more compact set of features along with high accuracy. The source code and testing datasets are available at https://github.com/jimy2020/SFLA_IWSSr-Feature-Selection.

## Introduction

The basic issue about big data is a large number of features. Among the features available, only a few of them will be useful to distinguish samples that belong to different classes and many of the features are irrelevant, noise, or redundant. Irrelevant features do not necessarily lead to noise generation in big data analysis; they result in increasing the dimensions of the dataset and computational complexity in clustering and classification operations, and consequently they decrease the rate of classification accuracy. Therefore, it is necessary to select the appropriate features. In feature selection, the redundant features are usually removed from dataset because there is a subset of other features that can provide the information that is provided by these redundant features. On the other hand, noise features that do not provide any information about labels should also be removed because they will reduce the efficiency of the algorithm. Therefore, only relevant features which consist of significant information about given dataset will remain^1^. Consequently, a method for identifying diverse features, calculating relationships between features and selecting relevant features is needed through a huge amount of data.

For a dataset containing *N* number of features, there are 2^*N*^ number of candidate subsets. The purpose of designing different feature selection methods has always been to find the most compressed subset with the highest precision among the candidate subsets. Considering the wide scope of possible solutions and increasing the size of this set of responses due to increment of the number of features exponentially, finding the best subset of N (medium or large) features is extremely costly. Computational complexity of selecting features is another major challenge for researchers^2^.

Different methods proposed for selecting a subset of features, have encountered some problems such as instability, high convergence time and falling in local optima as a final result, etc. Despite the success they have gained, they have not been able to extract the most effective features.

The feature selection methods are generally divided into four categories: filter methods, wrapper methods, hybrid methods and embedded methods. Each of these methods is described in detail^3^.

The filter methods for selection a subset of proper features use intrinsic and statistical characteristics of the features and they are independent of any learning algorithm. In these methods, weight is assigned to each feature based on the degree of relevance of features to class labels; correlation criteria and information theory-based criteria are used for weighting features usually. Due to the need for less computations, filter methods are effective for high-dimensional datasets, but they do not have the proper accuracy^4^. The filter methods are divided into two groups of univariate and multivariate. In univariate methods, relevance of only one feature is measured according to the evaluation criterion. In these methods, dependencies between features do not play a role in the process of feature selection. The methods are such as: t-statistics (TS)^5^, Signal-to-Noise Ratio (SNR)^6^, and Pearson Correlation coefficient (PC)^7^ and F-Test (FT)^8^. In multivariate methods, the relationship between the features is considered. This makes these methods slower than univariate methods. The methods are such as: minimum Redundancy Maximum Relevance (mRMR)^9^, Correlation based Feature Selection (CFS)^10^, Fast Correlation Based Filter (FCBF)^11^ and Mutual Information Feature Selection (MIFS)^12^, Max-Relevance-Max-Distance (MRMD)^13^, Analysis of variance (ANOVA)^14^ and F-Score^15^. In these methods, the features are sorted based on their weights, and the features that have higher weights are selected as relevant features.

MRMD method considers distance between two kinds of features. This method is based on distance function to measure the independence of every feature. The higher the distance, the more the independence. Therefore, Pearson’s correlation coefficient is used to measure the relevance between features. Distance functions such Euclidean distance, Cosine distance and Tanimoto distance are exploited to calculate the redundancy.

In ANOVA, a method is proposed to improve the prediction accuracy of mitochondrial proteins of the malaria parasite. In this method, firstly, the protein samples are formulated using the *g*-gap dipeptide composition. Then, Analysis of variance is proposed to select the best subset of features. Finally, the support vector machine (SVM) is used to perform the prediction.

The most important defect of filter algorithms is the lack of utilization of the classification accuracy in selection of a subset of features. To solve this problem, new methods called wrapper methods are proposed. Wrapper methods use learning algorithms and a classifier to find a subset of features. In these methods, the learning model has the tasks of searching in the space of primary features and selecting the subset of the candidate features.

Also, the classifier is used to estimate the performance of the subset of the selected candidate features. Compared to the filter methods, the wrapper methods have higher computational costs and they are not suitable for high-dimensional datasets; however, they are more successful in finding the subset of effective features and the high accuracy of selecting a subset of features using these methods is noticeable^16^. Many of wrapper methods have used heuristic search algorithms to find a subset of features. These methods start with a randomly generated solution, and in each iteration they are one step closer to the best subset of the solution. The evolutionary algorithms used in wrapper methods include Genetic Algorithm^17–19^, Simulated Annealing algorithm^20,21^, Ant Colony Optimization algorithm^22–24^, Shuffled Frog Leaping Algorithm^25,26^, Particle Swarm Optimization algorithm^27,28^, Binary Wolf Search Algorithm^29,30^ and so on.

Some methods use exhaustive searches. In^31^, in order to select a subset of features, it first starts with a complete set of features, and then some of the features are removed by the first depth method. In^32^, the features are selected using the beam search. This method arranges the features in a queue based on importance, and then all possible states are evaluated using beam searches. The main drawback of these methods is their computational complexity. Heuristic methods were proposed to solve this problem. Sequential feature selection methods such as Sequential Backward Selection (SBS)^33^ and Sequential Forward Selection (SFS)^34^, greedy methods such as hill-climbing^35^, Bayesian search methods such as Bayesian features selection^36^, meta-heuristic methods such as Ant Colony Optimization algorithm^22^, and Genetic Algorithm^17^, etc. are some methods that use heuristic search.

Another category of feature selection methods is Hybrid methods that combine filter and wrapper methods. So in the first step, based on a filter method, some features are selected based on importance. Then, in the selected features space, a wrapper method is applied to select the effective features^37–39^. In^40^, Incremental Wrapper Subset Selection (IWSS) is presented. In this method, after the weight of the features in the filter phase is calculated, the incremental algorithm is used to select the subset of features. First, the feature subset is empty. In the first iteration, the features are added with greater weights to the subset of features and the classifier is created based on the features and the dataset. The accuracy rate of classifier is stored as the best result. In next iterations, each time a feature with more weight is added to the subset, again the classifier is trained. If the recognition rate of the classifier is better than the one stored, the added feature is considered as a relevant feature and it is retained in the subset, otherwise the feature is removed from the subset. In^41^, the hybrid local search strategy embedded in the particle swarm optimization algorithm has been used to select relevant features. The purpose of the local search in this method is to optimize the particle swarm to select distinctive features based on their correlation information.

In^37^, a hybrid approach based on the Greedy Randomized Adaptive Search Procedure is proposed. In the first step, by using a filter method, the process of ranking the features is done, and features that have high degrees of relation with class labels are more weighty and less important features are less weighty. In the second stage, in the wrapper method, GRASP method is used to find the best subset. In the GRASP method, a subset of features is randomly selected based on their weights. Then in the next step by using the IWSS, SFS, IWSS, IWSSr, Hill-Climbing, and Best Agglomerative Ranked Subset (BARS)^42^ methods; redundant and irrelevant features are removed. Use of an improvement phase is also considered in FICA^43^. In this paper, at the filter step, features weighting is performed. Then, in the wrapper phase, using the Fuzzy Imperialist Competitive Algorithm (FICA) and the IWSSr algorithm, searching for effective features in the weighted feature space is done. In the other work, by using mutual information and adaptive genetic algorithm, gene expression data are classified^44^. In this method, the features are ranked base on maximizing the mutual information and then, by using the adaptive genetic algorithm, the optimal subset of features is selected. In^45^, an effective hybrid gene selection method based on ReliefF and Ant colony optimization (ACO) algorithm for tumor classification is proposed. At first, ReliefF is used to estimate the weights of features according to how well their values distinguish between close instances. Then a new pruning rule based on ACO is designed to reduce dimensionality and obtain a new candidate subset with the smaller number of genes.

A two-step feature selection is proposed to exclude redundant and noise information for identifying origin of replication in Saccharomyces cerevisiae. In this method, at first, the weight of the features is calculated based on the F-score technique. Then, the MRMR technique is used to maximize the correlation between features and class labels while minimize the correlation between features and features^46^.

In the embedded methods, selecting the features subset is considered as a part of the model construction. This kind of methods can be considered as a search in the feature and model space; such as Adaboost^47^, random forest, and decision tree^48^. SVM-RFE is also one of the embedded methods^49^. In this method, the algorithm starts with a set containing all features. In each iteration, the weight vector coefficients w is used to evaluate the features. Each element of this vector corresponds to a feature. In this case, the feature with the lowest score, ie, c_i_ = (w_i_)^2^, is removed. These weights indicate the relation of each feature with class label. Another algorithm proposed in this field is the KP-SVM algorithm^50,51^. The algorithm tries to find the appropriate features by updating the parameter σ in the RBF kernel.

In this paper, a hybrid method is proposed for selecting features in high dimensional datasets. In the proposed method, in the filter phase, the Relief method is used for weighting the features. Then, in the wrapper step, by using the SFLA and the IWSSr algorithm^52^, the search is performed to find the best subset of the features. The proposed method is evaluated with ten standard gene expression datasets. The results of the experiments confirm the effectiveness of the proposed approach in comparison with similar methods, in terms of Accuracy, Specificity, Sensitivity, Balance Rate and accessing to a subset of more compact features. The rest of the paper is organized as follows. Section 2 and 3 present an overview of the SFLA and IWSSr approaches and Section 4 describes the phases of the proposed method in detail. Section 5 provides the results of the method in the *gene* datasets. Finally, Section 6 summarizes the results.

## An Overview of the SFLA

SFLA is a new population-based metaheuristic optimization method that imitates the memetic evolution of a group of frogs when looking for a place with the maximum amount of available food. The SFLA has both definite and random strategies in finding the optimal response. The definite strategy allows the algorithm to use surface-level information efficiently in order to guide heuristic search. Random elements control the flexibility and power of the search pattern in the proposed method.

Inthis method, each frog is considered as a solution to the problem and a bunch of frogs forms a population that moves in order to reach a specific target. During the process of reaching the optimal answer, the population is divided into a number of subsets. The effects of the frogs in each subgroup modify the decision variables. After a certain number of evolutions, information is transmitted between the frogs during the process of combining subsets and forming a new population and a targeted search is carried out to determine the optimal answer. This trend continues until certain convergence conditions are established^53,54^.

In the SFLA, a primitive population of *sfla_p* frogs is randomly generated from possible answers. The position or situation of a frog is a possible solution to the problem. These frogs are implemented by vectors and structures to indicate the variables or problem solutions. In the algorithm, the entire initial population is first divided into sfla_m groups called memplex. Different memplexes that have *sfla_n* frogs are bunch of frogs that are individually searching for a solution in the search space. In each memplex, a submemplex is created to avoid falling in local optima^23^. Each submemplex consists of *sfla_q* frogs and the frogs are selected randomly based on the following probability function:

1 $${P}_{j}=\frac{2({\rm{sfla}}\_{\rm{n}}+1-{\rm{j}})}{{\rm{sfla}}\_{\rm{n}}\,({\rm{sfla}}\_{\rm{n}}+1)},\,j=1,2,\ldots ,sfla\_n$$

Where *P*_*j*_ is the probability of choosing jth frog for selection and *sfla_n* is the number of frogs in the memplex. Since in each memplex the frogs are sorted according to a descending order of fitness, by decreasing the fitness value, the probability of selecting frogs is lowered. Therefore, a better-positioned frog in the search space will have a greater chance of choosing as a member of the submemplex. In each submemplex, the worst frog (*P*_*w*_), performs leaping based on its own experiences and the position of best frog in memplex (*P*_*b*_). Therefore, the worst frog is first selected from the submemplex. The leaping step size for frog *P*_*w*_ is as follows:

2 $${{\rm{S}}}_{{\rm{B}}}=\{\begin{array}{cc}\min \{{\rm{int}}({\rm{rand}}.[{{\rm{P}}}_{{\rm{b}}}-{{\rm{P}}}_{{\rm{w}}}]).\,{{\rm{S}}}_{{\rm{\max }}}\} & {\rm{for}}\,{\rm{a}}\,{\rm{positive}}\,{\rm{step}}\\ \max \{{\rm{int}}({\rm{rand}}.[{{\rm{P}}}_{{\rm{b}}}-{{\rm{P}}}_{{\rm{w}}}]).-{{\rm{S}}}_{{\rm{\max }}}\} & {\rm{for}}\,{\rm{a}}\,{\rm{nagative}}\,{\rm{step}}\end{array}$$

Where *rand* is a random number in the range [0,1] and *S_max* is the maximum leap length. In the next step, the worst frog position is edited by the following equation:

3 $${{\rm{P}}}_{{\rm{w}}}^{\text{'}}={{\rm{P}}}_{{\rm{w}}}+{{\rm{S}}}_{{\rm{B}}}$$

If the new frog (${P}_{w}^{\text{'}}$) is better than the original frog, this frog is replaced with the original frog, otherwise the *P*_*w*_ frog is edited according to the best frog of the total population (*P*_*G*_) according to the following:

4 $${{\rm{S}}}_{{\rm{G}}}=\{\begin{array}{cc}\min \{{\rm{int}}({\rm{rand}}.[{{\rm{P}}}_{{\rm{G}}}-{{\rm{P}}}_{{\rm{w}}}]).\,{{\rm{S}}}_{{\rm{\max }}}\} & {\rm{for}}\,{\rm{a}}\,{\rm{positive}}\,{\rm{step}}\\ \max \{{\rm{int}}({\rm{rand}}.[{{\rm{P}}}_{{\rm{G}}}-{{\rm{P}}}_{{\rm{w}}}]).-{{\rm{S}}}_{{\rm{\max }}}\} & {\rm{for}}\,{\rm{a}}\,{\rm{nagative}}\,{\rm{step}}\end{array}$$

5 $${P^{\prime\prime} }_{w}={P}_{w}+{S}_{G}$$

**Figure 1:**
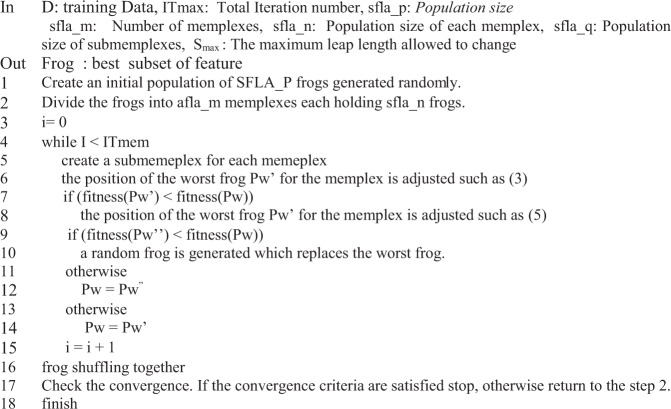
Pseudo code of SFLA.

Similar to the previous one, if the ${P^{\prime\prime} }_{w}$ frog is better than the original frog (*P*_*w*_), this frog is replaced with the ${P^{\prime\prime} }_{w}$ frog and if neither of these is satisfied, a new random frog is replaced with the worst frog of submemplex. After the *IT*_*mem*_ steps of dividing memplex into submemplexes, again all the frogs are combined and re-divided into *sfla_m* memplexes. This operation continues to meet the end conditions of the program. The pseudo code of SFLA is shown in Fig. 1. Based on this algorithm, the worst frog can leap toward the best frog. By repeating this process, gradually the average fitness of the frog population increases during the evolutionary stages and converges to a certain degree. With respect to this process, *P*_*G*_ and *P*_*w*_ are changed in each iteration and the value of fitness increases to converge to the desired response^55^.

## An Overview of IWSSr Algorithm

IWSSr algorithm^52^ that is an extension of IWSS algorithm, is one of the wrapper-based features subset selection algorithms. In this method, first, in the filter phase, the relevance of each feature with the class labels is calculated and a weight is assigned to each feature. In IWSSr, the SU criterion is used for weighting features. SU is a nonlinear information theory based criterion. This criterion evaluates each feature independently and it assigns to each feature a number in the range [0,1] indicating the weight of each feature based on its relevance to class labels. A large number indicates the high importance of the feature. This criterion is calculated as follows:

6 $$S{U}_{i,c}({F}_{i},C)=2\frac{H({F}_{i})-H({F}_{i}|C)}{H({F}_{i})-H(C)}$$

**Figure 2:**
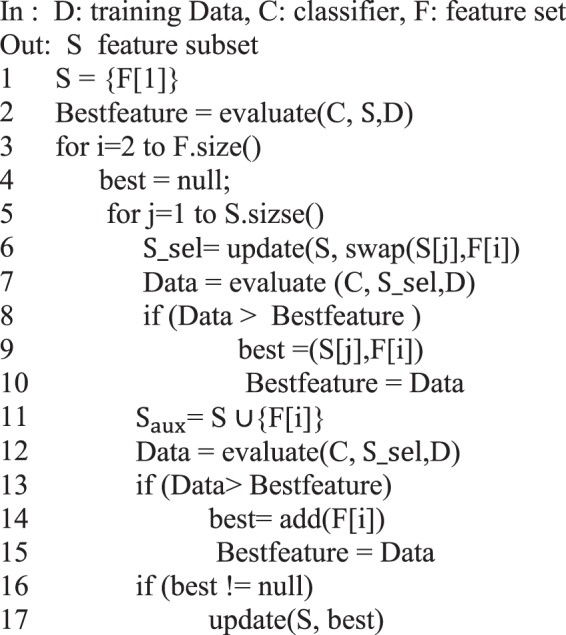
Pseudo code of IWSSr.

Where *C* is the class label, *F*_*i*_ represents ith feature and *H* indicates entropy. In the following, at wrapper phase, the features are arranged in descending order by weights. Then an incremental mechanism is used to select a subset of features. Figure 2 shows the pseudo code of IWSSr algorithm. In this algorithm, *S* is the subset of selected features. At first, the candidate subset is empty and in first iteration, the feature that has the highest score is added to the candidate subset.

Then a classifier is trained based on the candidate subset and the existing training data. The classification accuracy is maintained as the best result. The next step is done in two phases. In the first phase, a feature with a high score that has not been evaluated yet, is replaced with each feature in the candidate subset. After each replacement, a new classifier is trained by using the obtained subset. then the classification accuracy is calculated. If the addition of a new feature causes increase in classification accuracy compared to the previous subset, the result is maintained as the best. In this way, the dependence of this feature with all previous selected features is measured and if it does not depend on any of the selected features, it will be added to the candidate subset.

In the second phase, the feature that is under review (the feature that was replaced with the features in the selected subset in the first phase) is added to the selected subset *S* (which was obtained in the previous stage) and a new classifier is trained based on the new subset and the classification accuracy is calculated. If the accuracy of the subset is higher than the accuracy of the candidate subset of the first phase, it is maintained as the best result. After the first and second phases, if we have achieved a better subset in each of these phases, the optimal subset is selected as the subset of this iteration and the feature is applied to the selected subset.

## Materials and Methods

The proposed algorithm is a feature selection system called IWSSr and Shuffled Frog Leaping Algorithm (IWSSr-SFLA). In this paper, a hybrid method is proposed for selecting features in high dimensional datasets. In the proposed method in the filter phase, the Relief method is used for weighting the features. Then, in the wrapping phase, by using the combination of Shuffled Frog Leaping Algorithm and the IWSSr algorithm, the search is performed to find the best subset of features.

**Figure 3:**
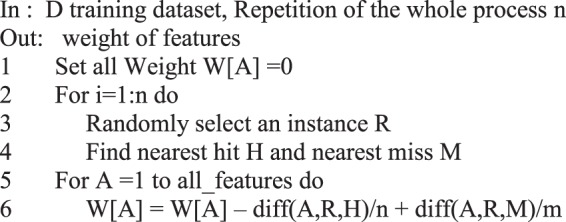
The general scheme of the Relief algorithm.

In the first phase, the Relief method, estimates the quality of features according to how well their values distinguish between instances that are near to each other. The Relief method calculates the correlation between features found by nearest-neighbor algorithm. Its output is a set containing weights of features^56^. It arranges the set in descending order. Figure 3 shows the general scheme of the Relief algorithm^56^.

As we can see in Fig. 3, at first, one sample is randomly selected, then its two neighbors are searched. One neighbor along with selected sample are in a same class and the other neighbor is in a different class. Function Diff(A,R,H) calculates the difference between the values of the feature *A* and the first neighbor, and Diff(A,R,M)) calculates the difference between the values of the feature *A* and the second neighbor. then the weight of each feature is updated. For discrete features the difference is 1 (when the values are different) and 0 (when the values are the same). For continuous features, the difference is the normalized value of the real difference of two values of feature, in the range of [0,1]. The Relief algorithm works well for noisy or correlated features. It depends on the number of features and the number of samples in the dataset. It is noticeable to point that the time complexity of the algorithm is linear.

In the wrapping phase, a primary population of frogs is initially created, each containing a subset of the features. In order to find the best subset for a more efficient classification, the primary population should be trained. After some learning phases, the best frog (which is closest to the target) is selected as a solution. At each training phase, the entire population is first divided into a number of memplexes.

In each memplex, a submemplex is selected and in this category the worst frog is initially trained or leaped towards the best frog of the memplex. If the better frog is created, this frog is replaced with the worst frog. Otherwise, the worst frog will be leaped according to the best frog of the entire set. This time, As the previous stage, if the frog is improved, it is replaced, and if not, a new frog is created. After creating the new frog randomly, the replacement of the new frog is done if its fitness is better than the original frog, otherwise the original frog is remaining unchanged. The division of the memplexes into submemplexes is repeated *IT*_*mem*_ times. After completing the learning phases, the whole set and the best frog get closer to the goal.

### Initial population creation

**Figure 4:**
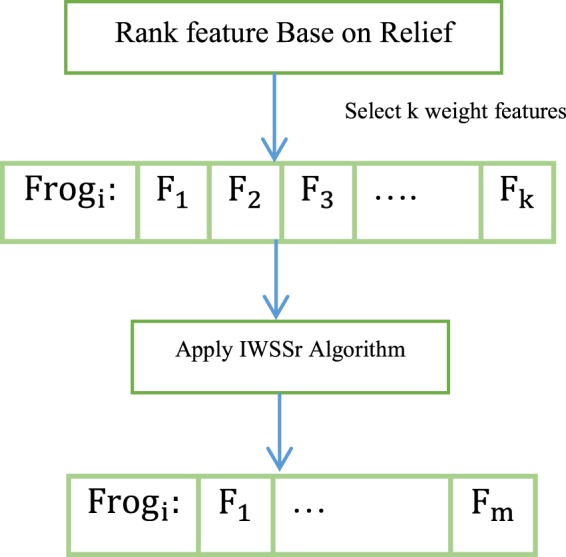
Create frogs in the proposed algorithm.

In the proposed algorithm, an Initial population with the number of *sfla_p* frogs is initially created randomly. Each frog has a subset of features for classifying data. Therefore any of the frogs will be a solution to the problem. In the initial population, a random percentage of the features are selected based on the weights assigned to them in the filtering phase. Due to random weighted selection, high weight features are more likely to be selected. Figure 4 shows how to create the frogs in the proposed algorithm.

### Evaluation of the initial population

After selecting the features for each frog, the reduntant features of each frog are removed by using the IWSSr algorithm and after applying this algorithm, the cost of each frog is calculated. The initial population is evaluated using a quality check function. The frog, which includes more relevant features, earns a higher value of fitness.

7 $${\rm{F}}=(\frac{{\rm{TP}}}{{\rm{TP}}+{\rm{FN}}}+\frac{{\rm{TN}}}{{\rm{TN}}+{\rm{FP}}})/2$$

Where *TN* is the number of negative samples which are correctly classified. *FN* is the number of positive samples identified as negative samples. *TP* is the number of positive samples which are correctly classified. *FP* is the number of negative samples identified as positive.

### Termination conditions of the program

The termination conditions refer to the user-defined conditions. The conditions can be a user-defined constant number of iterations for training, reaching the maximum percentage of diagnosis or not changing the entire population. In the experiments, after *IT*_*max*_ iterations, the learning process is terminated.

### Division of memplexes into submemplexes

In each memplex which has *sfla_n* frogs, a submemplex is created that contains *sfla_q* frogs. To do this, frogs of memplex are sorted by descending value of fitness. The probability of choosing each frog in submemplex is calculated based on Eq. (1). Therefore, the submemplex is created based on the fitness of each frog.

### Leap or improve the frog

**Figure 5:**
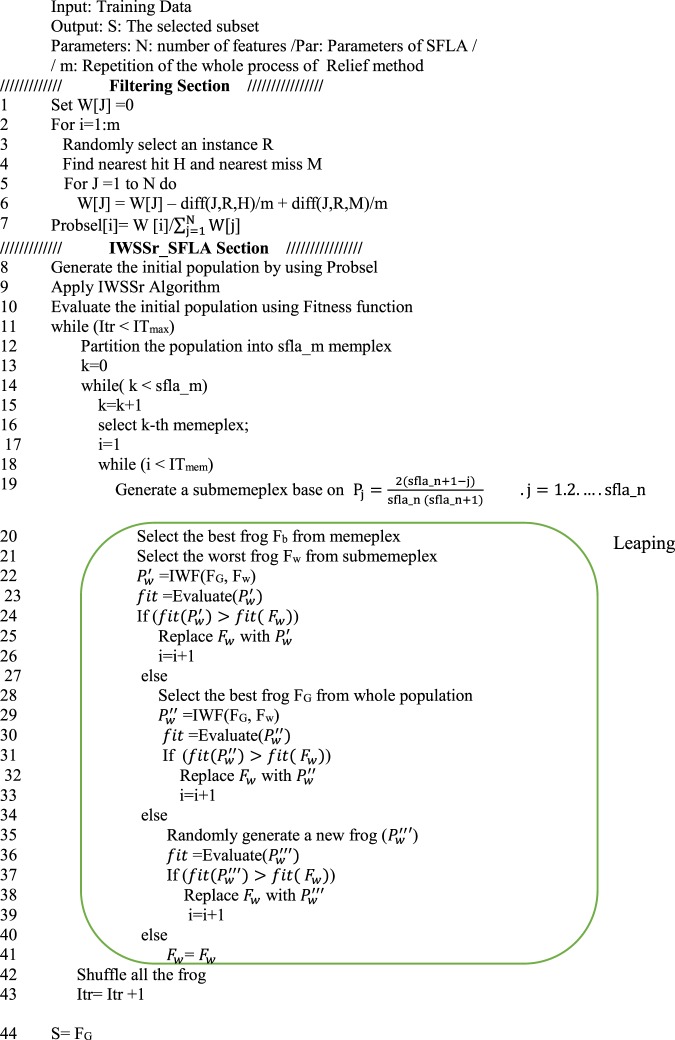
Pseudo code of the proposed hybrid algorithm.

**Figure 6:**
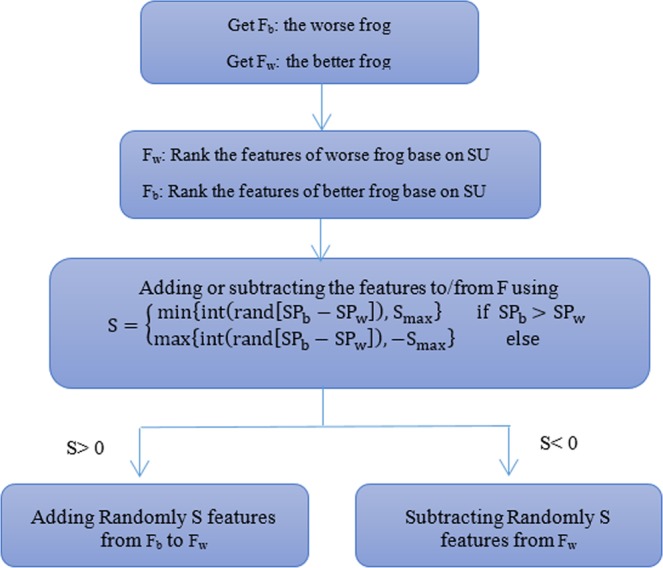
Leap algorithm for worst frog Improvement (F_w_) by the help of better frog (F_b_) (IWF).

After each submemplex creation, the worst frog position (*P*_*w*_) is edited based on the position of the best frog of the memplex (*P*_*b*_) (or the best frog of the total population (*P*_*G*_)). This edition is called leaping. Therefore, the leaping in the SLFA is an operation in which, the frog with a lower fitness can be improved according to a frog which has better fitness. The leaping action can vary depending on different issues. The improvement phase of the worst frog which is indicated by the IWF as shown in Fig. 5, is illustrated as a flowchart in Fig. 6.

To improve the worst frog (*P*_*w*_) according to better frog in the memplex (*P*_*b*_), at first, the number of features that are removed from or added to the frog is calculated using the following equation:

8 $${{\rm{S}}}_{{\rm{b}}}=\{\begin{array}{cc}\min \{{\rm{int}}({\rm{rand}}[{{\rm{SP}}}_{{\rm{b}}}-{{\rm{SP}}}_{{\rm{w}}}]),{{\rm{S}}}_{{\rm{\max }}}\} & {\rm{if}}\,{{\rm{SP}}}_{{\rm{b}}} > {{\rm{SP}}}_{{\rm{w}}}\\ \max \{{\rm{int}}({\rm{rand}}[{{\rm{SP}}}_{{\rm{b}}}-{{\rm{SP}}}_{{\rm{w}}}]),-{{\rm{S}}}_{{\rm{\max }}}\} & {\rm{else}}\end{array}$$

Where *SP*_*w*_ and *SP*_*b*_ are the number of features in the worst and better frogs respectively. *rand* is a random number in the range of [0,1] and *S*_*max*_ is the maximum number of feature changes allowed. In order to make changes in the worst frog, at first, according to the SU criterion, the features of the worst and better frogs are arranged. Then, if *S*_*b*_ is a positive number, then *S*_*b*_ features are randomly added to the worst frog from the better frog. In this case, the features that have high weights are more likely to be selected. Similarly, if *S*_*b*_is negative, then *S*_*b*_ features are randomly deleted from the worst frog. In this case, features that are less weighted are more likely to be selected. In the next step, by using the IWSSr algorithm, the reduntant features of the worst frogs are removed.

## Results and Discussions

### Datasets

In order to evaluate the proposed method, the experiments are performed by MATLAB software on ten gene expression datasets. Summary of the datasets are given in Table 1. Each dataset is descripted as follows:

**Table 1:** Microarray data sets used in the experiments.

Data set	Original Data	Training Data	Independent Data	#Gene	#Classes	#class1	#class2
Colon	62	50	12	2000	2	40	22
Arcene	100	80	20	10000	2	44	56
Prostate1	88	71	17	12625	2	38	50
DLBCL	77	61	16	11226	2	58	19
Lung	181	145	36	12533	2	150	31
Dorothea	800	640	160	100000	2	610	190
Prostate	136	109	27	12600	2	77	59
CNS	60	48	12	7129	2	21	39
Leukemia	72	58	14	7129	2	47	25
Breast	97	78	19	24481	2	51	46

Prostate dataset: This dataset contains 12600 genes for 136 samples. 77 samples include prostate tumor and 59 samples are normal^57^. Colon dataset: This dataset contains 2000 genes and 62 samples. 40 samples contain colon cancer and 22 samples are normal^58^. Central Nervous System dataset (CNS): This dataset contains 7129 genes and 60 samples. The dataset includes 21 benign samples and 39 malignant samples^59^. Dfiuse Large b-cell lymphoma dataset (DLBCL): This dataset contains 11226 genes for 77 samples. 58 samples including lymphoma tissue, are large cell B, and 19 samples of lymphoma tissue are Follicular lymphoma^60^. Dorothea dataset: This dataset contains 100,000 features and 800 samples. 190 samples are positive and 610 are negative^57^. Leukemia dataset: This dataset contains 7129 genes and 72 samples. Diseases of the leukemia collection are divided into two categories of Acute Lymphoblastic Leukemia (ALL) and Acute Myelogenous Leukemia (AML). The dataset consists of 47 ALL samples and 25 AML samples^61^. Arcene dataset: This dataset contains 10,000 genes and 100 samples. This dataset consists of 56 cancer samples and 44 normal samples^57^. Lung cancer: Gene expression dataset for lung cancer classification between two classes: adenocarcinoma (ADCA); malignant pleural mesothe-lioma (MPM). The lung dataset contains 181 tissue samples (150 ADCA and 31 MPM). Each sample is described by 12533 genes^62^. Breast cancer: Patients outcome prediction for breast cancer. The training data contains 97 patient samples, 46 of which are from patients who had developed distance metastases within 5 years (labelled as “relapse”), the rest 51 samples are from patients who remained healthy from the disease after their initial diagnosis for interval of at least 5 years (labelled as “non-relapse”). In this data,the number of genes are 24481^57^. Prostate1 dataset: This dataset contains expression levels of 12625 genes taken over 88 samples. (38 normal samples and 50 abnormal)^63^.

### Performance metrics

To compare the results of the proposed method, seven hybrid methods LFS, IWSS, IWSSr, BARS, GRASP, SVM-RFE and FICA and three filter methods FCBF^24^, F-Score and PCA^51^ have been used. The PCA method has been proposed for high-dimensional datasets in recent years. To demonstrate the performance of the proposed method some metrics such as, the number of features obtained, the number of evaluations performed to reach the final subset, accuracy, specificity, sensitivity, and balance rate according to the following formula are measured^64,65^. The number of evaluations indicates the number of subsets tested to reach the final subset.

9 $${\rm{Accuracy}}=\frac{TP+TN}{TP+TN+FP+FN}$$

10 $${\rm{Specificity}}=\frac{TN}{TN+FP}$$

11 $${\rm{Sensitivity}}=\frac{TP\,}{TP+FN}$$

12 $${\rm{BR}}=\frac{{\rm{Specificity}}+{\rm{Sensitivity}}\,}{2}$$

The classifier used in the proposed method is support vector machine and in the methods to be compared, Bayesian classifier is used.

When using feature selection methods, it is important to make sure that there is no overlap between the training and test data. Cross validation is an approach that puts data into categories effectively to evaluate feature selection and classification methods. In this approach, the efficiency of the proposed methods is evaluated on the basis of a number of categories derived from the original data. At first, the whole samples of a dataset are randomly divided into k categories for training and testing purposes. In k steps, (k-1) batches are used for model training and one batch is used for testing. At each step, the features and parameters used to test the model are obtained from the training stage and with the help of samples in the training categories. Finally, the efficiency of the proposed method is obtained based on the k outputs of the training and testing phases^66,67^.

In this paper, Cross Validation (CV) method is used to train and then test the support vector machine classifier based on selected features to determine the percentage of recognition of test data, where k = 10. Since in the 10-fold CV method, the samples are randomly divided into 10 categories, the results depend on how the samples are grouped. To solve this problem, the samples are randomly divided into 10 groups 10 times.

The final number of features is equal to the average of selected features and other criteria are equal to the average of the criteria in selected subset after 10 times execution of proposed method. The performance criteria of the proposed method is also obtained based on the average of 10-fold CV repetitions.

The initial value of hyper parameters of the proposed method is given in Table 2. All hyper parameters are selected based on multiple tests and they are identical in all datasets. To determine the value of hyper parameters, the Random search method is used. For this purpose, a set of hyper parameters is chosen and the model is built based on training data and then it is evaluated based on evaluation data. This process is repeated with other hyper parameters. The hyper parameters that report the best accuracy are selected. In this paper, Population size is set from 80 to 120, Number of memplexes is set from 8 to 12, Population size of submemplexes is set from 3 to 6. The maximum leap length allowed to change (S_max_) is set from 3 to 8.

**Table 2:** SFLA parameters used in the problem.

Parameter	Value	Comments
*sfla_p*	100	*Population size*
*sfla_m*	10	Number of memplexes
*sfla_n*	10	Population size of each memplex
*sfla_q*	4	Population size of submemplexes
*IT*_*max*_	40	Total Iteration number
*IT*_*mem*_	10	The number of replications of the division of memplexes into submemplexes
*S*_*max*_	5	The maximum leap length allowed to change

### Experimental results

In Tables 3 and 4, the results of the implementation of the proposed method have been shown along with comparative methods. In this following tables, acc refers to the accuracy and atts refers to the attribute. According to Table 3, the results approve that the BARS method has fewer features and better accuracy than other methods.

**Table 3:** Result of feature selection algorithm.

DataSet	IWSS		IWSSr		LFS		BARS		FCBF		PCA	
	Acc	Atts	Acc	Atts	Acc	Atts	Acc	Atts	Acc	Atts	Acc	Atts
Colon	80.65	3.8	83.87	2.8	80.80	4.1	85.70	3.0	77.40	14.6	72.50	28.9
Arcene	70.00	13.4	72.00	6.2	73.00	4.5	74.00	4.9	70.00	34.2	—	—
Prostate1	76.23	12.8	77.42	8.3	73.12	3.6	85.34	4.1	63.12	32.4	59.12	37.1
DLBCL	83.11	3.2	81.23	2.7	88.67	4.1	75.21	2.8	96.45	56.2	68.11	42.7
Lung	97.20	2.7	97.20	2.4	93.60	2.5	98.30	3.0	99.40	115.2	85.61	125.2
Dorothea	93.50	7.4	92.90	6.3	90.30	5.5	93.80	7.3	92.60	92.8	—	—
Prostate	77.90	11.1	78.70	7.0	75.40	4.5	86.80	3.7	61.30	35.8	57.35	36.6
CNS	85.21	3.2	86.10	3.1	83.23	3.4	89.12	2.8	93.24	42.2	77.32	44.1
Leukemia	87.50	2.5	87.50	3.0	93.00	3.2	90.50	2.3	95.80	45.8	79.10	53.8
Breast	69.21	11.1	70.21	9.2	70.43	10.1	72.81	9.34	69.43	107.3	63.10	96.3
**Mean**	**82.05**	**7.122**	**82.71**	**5.1**	**82.15**	**4.55**	**85.15**	**4.32**	**81.87**	**57.65**	**70.27**	**58.09**

**Table 4:** Comparison of proposed method with GRASP and FICA.

	Grasp + HC		Grasp + IWSS		Grasp + IWSSr		Grasp + BARS		Grasp + SFS		FICA + IWSSr		F-Score		SVM-RFE		Proposed method	
	Acc	Atts	Acc	Atts	Acc	Atts	Acc	Atts	Acc	Atts	Acc	Atts	Acc	Atts	Acc	Atts	Acc	Atts
Colon	81.10	3.0	79.60	3.4	82.20	3.1	80.00	2.9	80.00	3.5	93.60	4.5	83.74	55	93.70	9.8	94.72	5.3
Arcene	80.00	5.7	79.30	6.0	78.50	5.7	79.00	5.2	79.30	6.3	93.40	7.1	73.25	110	89.11	13.5	95.16	8.5
Prostate1	80.45	4.3	79.12	4.1	78.49	3.7	81.12	4.7	78.43	6.3	—	—	68.74	105	82.71	17.2	88.52	8.2
DLBCL	85.65	2.1	84.60	2.2	85.61	2.1	89.11	2.2	85.70	2.4	99.10	4.5	93.11	100	95.23	15.7	99.21	6.8
Lung	95.60	2.2	95.08	2.2	95.70	2.4	96.02	2.3	96.20	2.4	98.90	3	82.16	105	98.73	9.4	99.16	5.6
Dorothea	93.30	3.7	93.30	4.2	92.90	3.8	93.50	5.0	93.20	4.4	75.80	3	76.24	310	84.32	21.7	91.43	7.2
Prostate	77.80	5.0	78.60	5.7	77.50	4.6	78.60	5.1	78.10	5.6	92.40	4.4	54.33	250	92.20	14.4	94.18	7.8
CNS	91.46	2.6	93.12	2.8	87.32	2.8	92.14	3.1	91.12	3.1	—	—	66.53	90	76.96	16.3	95.64	6.7
Leukemia	92.60	2.7	93.70	2.7	91.60	2.8	93.30	2.8	93.60	3.3	99.60	1.8	75.57	70	100.00	8.6	99.62	5.2
Breast	79.63	4.3	80.11	3.1	78.38	3.5	81.24	2.7	80.91	3.6	—	—	73.82	120	86.09	17.3	88.17	10.2
Mean	85.75	3.56	85.65	3.64	84.82	3.45	86.40	3.60	85.65	4.09	—	—	74.74	131.5	89.90	14.39	93.34	7.12

The main idea behind this approach is based on relevancy and redundancy; so the features are added to the selected set that have better information for the classification of the data. The results show that the LFS^5^ method has fewer features, but does not have good accuracy. Due to the use of only 100 filtered features to select the subset of features in the wrapper phase, the relationship between the features cannot be considered. The IWSS and IWSSr are wrapper methods. Although the IWSS method finds the subset fast because of relying on the univariate ranking of features, does not consider the relationship between the features. It often fails to find redundant features and the average number of features found by this method is high. In the IWSSr method, in each step of the implementation, the dependence of the assessed feature with all of the features in the selected subset is examined.

Therefore, in addition to the high accuracy, it finds a subset of more compact features in comparison with the IWSS method. However, this method requires a high evaluation time compared to similar methods and runs slow on high dimensional datasets. FCBF and PCA methods are filter-based. These methods only consider the linear relationship between features to find irrelevant features, so they cannot remove the redundant features, and the number of features found in these methods is high.

In Table 4 the proposed method is compared with Grasp, IFCA, F-score and SVM-RFE. In the Grasp method, after finding the candidate subsets, in the local search phase, the methods of IWSS, IWSSr, SFS, BARS, and Hill Climing are used separately to select the best subset of features. The BARS method selects the best subset of features using a combination of candidate subsets of features and removing the redundant features. The GRASP method, using a two-step algorithm as well as the application of various techniques in the improving phase section, has made progresses in comparison with other methods. However, it is less efficient than the proposed method and FICA. FICA method, because of using the IWSSr method, considers the relationships between features. The Fuzzy Imperialist Competitive Algorithm has been able to remove redundant features properly.

Additionally, the fuzzy influence of imperialist in colonies and the distribution of relevant features of imperialists in the colonial subsets leads to select the subset of optimal features with high-performance. Although this method finds a subset of more compact features than the proposed method, the results show that the accuracy of this method is competitive with the proposed method.

The F-score method is usually utilized to compute the degree of difference between two sets of real numbers. The larger the F value, the better the predictive ability of the feature^68^. In this study, F value for all features is calculated in the datasets. Then, the 55 high F values are selected for classification using SVM. Although this method is simple, it’s detection rate is lower than the proposed method. This method does not indicate mutual information of features. In other words, F-score reveals the discriminative power of each feature independently from other features. Also, the number of selected features in this method is much higher than the other methods.

The SVM-RFE method (Support Vector Machine based on Recursive Feature Elimination) ranks the genes by training a SVM model and selects important genes using recursive feature elimination strategy. In this method, RFE is applied for eliminating unimportant features^69^. Therefore, firstly, the SVM training using initial set of features is performed and the weight is assigned to each feature. Then, these absolute weights are sorted in descending order. Finally, the less weighted features are deleted. The results show that accuracy rate of this method is appropriate, but, the main problem of SVM-RFE is its time complexity, especially when the dimensionality of input data is extremely high. Furthermore, the number of selected genes in this method is higher than the other methods.

The results show that the accuracy of the proposed method in all datasets except Dorothea, is better than other methods. First, it is able to remove irrelevant features in the filter phase, then it removes the redundant features from the subset of features using the hybrid of the SFLA and IWSSr. In this method, due to the improvement of worst frogs, based on better frogs in the memplex and the best frog in the whole set, the redundant and irrelevant features of the frogs are removed and the relevant and useful features are added to the frogs. Removing and adding features are done based on their importance and their relationship with each other. Therefore, the selected feature set is more compact in the best frog and includes relevant features. The results show that in 8 datasets of 10 datasets used, an accuracy of 90% and in 3 datasets, a high accuracy of 98% is achieved. In 10 datasets used in the proposed method the average accuracy of 93.34% is obtained that is better than what obtained from other methods. Additionally, the average of selected features is 7.12, that can be compared to other methods.

In order to better evaluation, in this study, each dataset is divided into two datasets; a training dataset and an independent dataset. 80% of the original data is chosen randomly for the training dataset and 20% for the independent dataset. For this purpose, the training dataset is used to train, evaluate and justify the proposed method, and the independent dataset is applied for final performance evaluation of the proposed method. The samples are randomly divided into 2 groups 10 times and the results are averaged over 10 times. The results of these experiments are shown in Table 5. The results approve that the proposed method is robust and it has high accuracy rate. Therefore, the method can be used to classify gene expression data with high accuracy.

**Table 5:** Performance results of proposed method in training and independent data.

	Training data				Independent data			
	Accuracy	Sensitivity	Specificity	Balance rates	Accuracy	Sensitivity	Specificity	Balance rates
Colon	94.50	95.87	86.11	90.99	93.33	95.00	90.00	92.50
Arcene	94.75	92.57	96.44	94.50	94.00	92.21	95.45	93.83
Prostate1	88.87	86.77	90.50	88.63	88.23	87.77	91.00	89.38
DLBCL	99.50	98.89	94.81	96.85	98.12	98.33	95.00	96.66
Lung	99.13	99.58	96.80	98.19	99.16	99.66	96.66	98.16
Dorothea	91.25	93.70	90.26	91.98	90.37	91.31	89.47	90.39
Prostate	94.18	98.36	90.45	94.41	94.44	96.25	91.81	94.03
CNS	95.31	90.54	97.21	93.88	94.99	92.50	96.25	94.37
Leukemia	99.34	100.00	97.29	98.64	98.57	99.00	97.50	98.25
Breast	88.12	88.23	87.95	88.09	87.89	89.00	85.55	87.27

**Figure 7:**
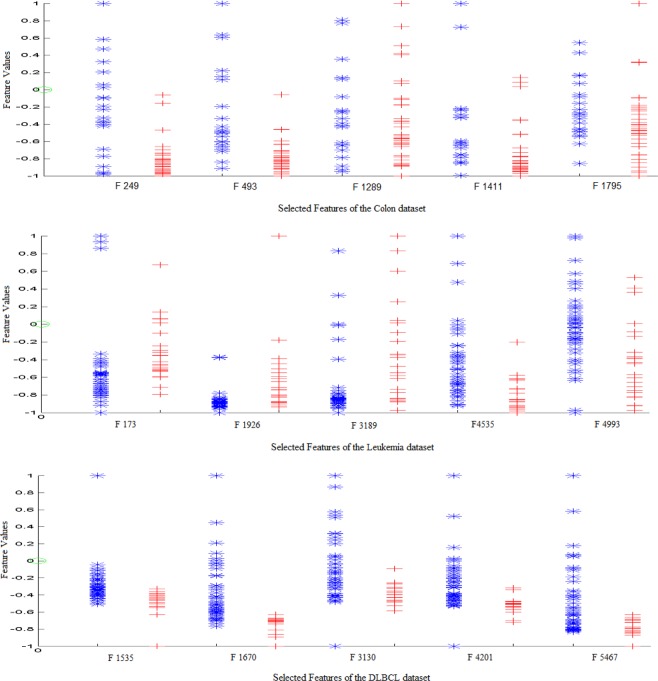
Distribution of selected feature values using the proposed method.

In addition, a more detailed analysis of the proposed method, focusing on the features selected, shows some interesting aspects. Figure 7 shows value of the selected features for all samples in some datasets. The proposed method has selected features whose values are less overlapping in the two classes. So these features have distinguished the patterns of two classes even better. It shows that the proposed method has selected appropriate features properly based on the available information. Also, the features in the negative class, especially in the DLBCL and Colon Datasets, have less variance. This property may be important in this regard that in the test and not seen samples, the value of the features is also in the range shown in the Fig. 7. Therefore, the error rate in this class can be less in comparison with other class. However, the value of features in the positive class has more variance. This causes the test data to deviate more than the mean, and the error rate in this class increases. Therefore, feature selection methods should select features that have a high classification accuracy on the test and training data.

**Figure 8:**
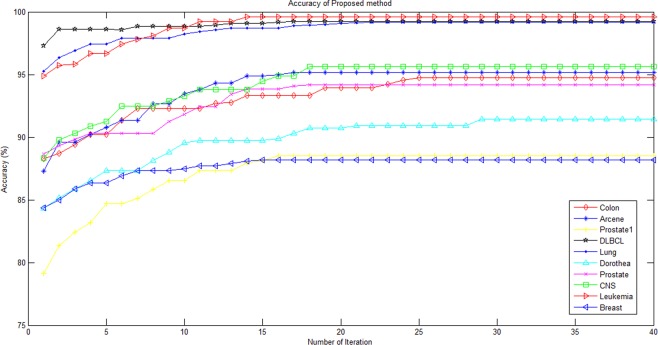
Mean accuracy of frog’s populations in the 40 iterations of training.

To study the process of convergence of the algorithm, the mean accuracy of the method on the datasets in 40 iterations is shown in Fig. 8. As you can see, the learning process is going fast at the beginning, on average in step 20, the algorithm has converged on most datasets, and the accuracy has not increased from this iteration.

Moreover, the minimum, maximum and average number of iterations to achieve convergence of the proposed algorithm using the datasets are shown in Table 6. The Arcene dataset with an average of 7.8 iterations has the lowest convergence time and Breast dataset with an average of 9.5 iterations has the highest convergence time. Overall, the average number of iterations required for all datasets is 12. 38 reps.

**Table 6:** minimum, maximum and average number of iterations performed by the proposed algorithm.

Dataset	Minimum number of iterations	average number of iterations	Maximum number of iterations	Average accuracy
Colon	12	13.9	18	94.72
Arcene	4	70.8	9	95.16
Prostate1	6	9.40	15	88.52
DLBCL	9	14.7	16	99.21
Lung	5	80.7	10	99.16
Dorothea	9	11.4	14	91.43
Prostate	17	22.2	30	94.18
CNS	8	9.80	21	95.64
Leukemia	11	16.4	19	99.62
Breast	12	9.50	22	88.17
Average	9.3	12.38	17.4	93.34

**Figure 9:**
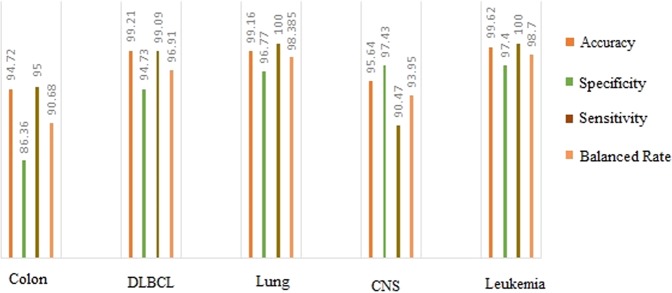
Comparing the performance criterion (accuracy, Specificity, Sensitivity and Balanced Rate) of proposed method.

By checking the number of samples in two classes of data it is clear that the number of data for two classes in Colon, DLBCL, Lung, CNS and Leukemia datasets is not balanced. In this type of data, the method cannot be evaluated only based on the “precision” criterion. Because the method may be biased to the majority class. In order to better evaluate, the accuracy, specificity, sensitivity and balance rates of proposed method in the mentioned datasets are shown in Fig. 9. Obviously, the proposed method has classified the class with more samples properly. However, the class with fewer samples has been classified with fewer classification rate. Due to the low number of samples in the class for correct learning, the classification operation is justifiable. Generally, all the criteria except Specificity in Colon dataset is higher than 90%. The results of the Fig. 9 show that the performance of proposed method in the classification of unbalanced data is also acceptable.

## Conclusion

In this paper, a two-step hybrid algorithm based on Shuffled Frog Leaping Algorithm is proposed. This method uses the advantages of filter and wrapping methods for selecting efficient features. In the filter phase of the proposed method, the Relief method is used for weighting the features of the dataset. Then, in wrapping phase, in the weighted space, by using the Shuffled Frog Leaping Algorithm and the IWSSr algorithm, the search is performed to find the effective and relevant features. In the phase of modifying frogs, removing and adding features are based on their importance and weight. Therefore, the proposed method detects the relationship between the features properly and removes the redundant and irrelevant features from the selected feature set. The proposed method is evaluated using ten gene standard datasets. The experimental results of the proposed algorithm approve that it has the highest accuracy (an average of 93.34%) in comparison with similar methods. Also, the number of features found in each dataset with an average of 7.12 causes high efficiency and a subset of compressed features is achieved.
